# Protective Roles of Apigenin Against Cardiometabolic Diseases: A Systematic Review

**DOI:** 10.3389/fnut.2022.875826

**Published:** 2022-04-15

**Authors:** Yajie Xu, Xue Li, Hui Wang

**Affiliations:** ^1^State Key Laboratory of Oncogenes and Related Genes, Center for Single-Cell Omics, School of Public Health, Shanghai Jiao Tong University School of Medicine, Shanghai, China; ^2^Shanghai Institute of Nutrition and Health, Chinese Academy of Sciences, Shanghai, China

**Keywords:** apigenin, flavonoid, cardiometabolic disease, metabolic syndrome, signaling pathways

## Abstract

Apigenin is a flavonoid with antioxidant, anti-inflammatory, and anti-apoptotic activity. In this study, the potential effects of apigenin on cardiometabolic diseases were investigated *in vivo* and *in vitro*. Potential signaling networks in different cell types induced by apigenin were identified, suggesting that the molecular mechanisms of apigenin in cardiometabolic diseases vary with cell types. Additionally, the mechanisms of apigenin-induced biological response in different cardiometabolic diseases were analyzed, including obesity, diabetes, hypertension and cardiovascular diseases. This review provides novel insights into the potential role of apigenin in cardiometabolic diseases.

## Introduction

**FIGURE 1:**
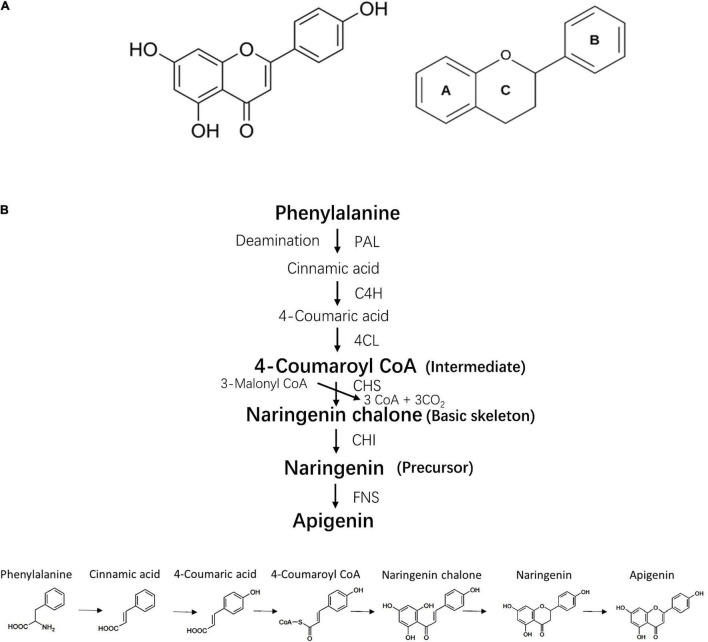
Chemical structure and bio-synthesis of apigenin. **(A)** Chemical structure of apigenin and its basic skeleton. **(B)** The process of apigenin bio-synthesis. PAL, phenylalanine ammonia lyase; 4CH, cinnamate 4-hydroxylase; 4CL, 4-coumaroyl CoA ligase; CHS, chalcone synthase; CHI, chalcone isomerase; FNS, flavone synthase.

Apigenin (4′,5,7-trihydroxyflavone) is named after the genus *Apium* belonging to family Apiaceae (1). It is widely distributed in vegetables and fruits, such as celery, parsley, oranges and garlic (2), and is also found in herbs such as snow lotus and chamomile (3) (Figure 1A). As a secondary plant metabolite, apigenin is usually stored in plants in a water-soluble glycosylated form (4). Purified apigenin is a yellow powder with a low molecular weight (MW 270.24). It is nearly insoluble in water, moderately soluble in hot alcohol and soluble in dimethyl sulfoxide (DMSO) (5). Pure apigenin is chemically unstable and therefore stored in the dark at −20°C (5).

The biosynthesis of apigenin occurs on the surface of the endoplasmic reticulum and requires four steps including intermediate synthesis, basic skeleton synthesis, precursor synthesis and generation of the apigenin structure (6) (Figure 1B). Current evidence indicates that the bioactivity of apigenin is dependent on its chemical structure, thus the structure-activity relationship of apigenin can be determined by extracting the molecular fragments associated with a specific biological activity. For example, double bonds in the two aromatic rings and hydroxyl groups on C-7 and C-4′ induce the inhibition of α-glucosidase and α-amylase (7). The C- 4′ hydroxyl group in ring B is essential for immunomodulatory properties (8). The hydroxyl radicals at position 5, 7 and 4′ are necessary for Liver X receptor activation (9).

Cardiometabolic disease links the metabolic syndrome disorders (abdominal adiposity, hypertension, dyslipidemia, hyperinsulinemia and glucose intolerance) that are predictive of cardiovascular disease and Type 2 diabetes (T2DM) (10). Recently, apigenin has been found to play a protective role in cardiometabolic diseases *in vitro* and *in vivo*. This systematic review summarizes the current perspective.

## Protective Roles of Apigenin in Cardiometabolic Diseases

### Protective Role of Apigenin in Obesity and Lipid Metabolism

Obesity is attributed to chronic energy imbalance, including excessive energy intake and limited energy expenditure (11, 12). Anti-obesity strategies focus on suppression of energy intake and stimulation of energy expenditure by regulating lipid metabolism, such as inhibiting pancreatic lipase activity and adipocyte differentiation (13). Studies suggest that apigenin controls energy intake by inhibiting appetite and stimulating energy expenditure by regulating lipid metabolism to alleviate obesity.

First, apigenin inhibits obesity by suppressing food consumption. *In vitro* and *in vivo* studies confirmed that apigenin upregulates the expression of anorexigenic neuropeptides pro-opiomelanocortin (POMC) and cocaine- and amphetamine-related transcript (CART), resulting in inhibition of food intake. N-29-2 and SH-SY5Y cells transfected with pPOMC-Luc and pCART-Luc vectors were treated with 0.2–5 μM apigenin for 6 h, resulting in upregulation of pPOMC-Luc and pCART-Luc activity. In an *in vivo* study, 6-week-old C57BL/6J mice were injected (every 24 h) intraperitoneally with 1 or 10 mg/kg of apigenin in a short-term intervention. Male C57BL/6J mice fed with a high-fat diet (HFD) or a standard laboratory chow diet received 0.05% apigenin for 30 days to demonstrate that apigenin reduces food intake and visceral fat over a long-term period (14). POMC and CART neurons found in the retro-chiasmatic area and throughout the rostrocaudal span of the arcuate nucleus (ARC) play a role in appetite control (15). Increasing expression of POMC and CART induces the expression of leptin receptor B (LepRb) to facilitate leptin binding to LepRb, resulting in an anorexic effect and upregulation of insulin receptors to inhibit appetite.

Secondly, apigenin stimulates energy expenditure by regulating lipid metabolism, including adipogenesis, lipolysis, fatty acid oxidation, and cholesterol synthesis. Recent studies indicate that adipose tissues are generally targeted by apigenin eliciting the following effects:

(1) *Stimulation of PPAR*γ *signaling*. Several studies have demonstrated that apigenin inhibits adipocyte differentiation *via* STAT3 (the signal transducer and activator of the transcription 3)-CD36-PPARγ (peroxisome proliferator-activated receptor-gamma) axis (16) and AMPK (5′-Adenosine monophosphate-activated protein kinase)/PPARγ axis (17). One study showed that 100 μM apigenin treatment inhibits the differentiation of 3T3-L1 preadipocytes to mature white adipocytes. Mouse models of diet-induced obesity receiving apigenin *via* subcutaneous injection for 13 days showed that apigenin reduced visceral fat mass (16). Apigenin binds to non-phosphorylated STAT3 to decrease STAT3 phosphorylation and nuclear translocation (18), followed by a decline in the expression of CD36, the downstream target gene involved in fatty acid transport (19). PPARγ is the transcript factor of central ligand-activated transcription factors. It inhibits adipogenesis and controls adipose tissue differentiation to regulate inflammation in obesity. PPARγ expression depends on CD36 expression and therefore apigenin treatment inhibits adipocyte differentiation *via* downregulation of PPARγ. Other studies reported that apigenin activates the phosphorylation of AMPK (5′-adenosine monophosphate-activated protein kinase) to downregulate adipogenesis *via* AMPK/PPARγ axis in 3T3-L1 cells treated with 10 μM apigenin for 1 h or 4 days (17) and in HFD mice treated with 200 mg/kg enzyme-treated celery extract (20). AMPK acts as a potential target against adipogenesis (21, 22) and downregulates the expression of PPARγ. The adipogenous genes downstream of PPARγ, such as fatty acid-binding protein 4 and stearoyl-CoA desaturase, are also downregulated, thereby suppressing adipogenesis (17).

(2) *Repression of enzyme activity.* Guo et al. reported that 0.6 mM apigenin directly inhibits pancreatic lipase activity *in vitro* (23). Pancreatic lipase catalyzes the conversion of triglycerides to monoglycerides and fatty acids in the intestine. Obesity is alleviated by the suppression of pancreatic lipase, fatty acid synthesis and fat absorption. Gómez-Zorita et al. showed that treatment with 25 μM apigenin decreases the expression of fatty acid synthase (FAS), while increasing the expression of adipose triglyceride lipase (ATGL) in mature adipocytes derived from human mesenchymal stem cells (hMSCs), resulting in reduced adipogenesis (24).

(3) *Activation of lipolysis-related genes*. Apigenin regulates lipolysis *via* activation of lipolysis-related genes. In a recent study, 3-week-old HFD mice (C57BL/6J, male) treated with 0.04% apigenin for 12 weeks showed upregulation of lipolysis-related genes in white adipose tissues (WAT), such as FOXO1 (Forkhead Box O1) and SIRT1 (Sirtuin 1) (25).

(4) *Induction of fatty acid oxidation*. Dietary apigenin induces phosphorylation of AMPK and 1-aminocyclopropane-1-carboxylic acid (ACC) in brown adipose tissues (BAT) to utilize free fatty acids synthesized from white adipose tissues (WAT) (25).

Liver, in addition to adipose tissues, is essential for lipid metabolism. Abnormal lipid metabolism in the liver induced by obesity may cause hepatic steatosis. Apigenin also improves lipid metabolism in the liver to alleviate hepatic steatosis *via* following mechanisms:

(1) *Stimulation of PPAR*γ *signaling*. Apigenin modulates PPARγ expression in hepatic lipid metabolism *via* Nrf2-PPARγ axis. In Hep1-6 cells, apigenin activates nuclear factor erythroid 2-related factor 2 (Nrf2) *via* translocation into the nucleus to upregulate downstream antioxidant enzymes and downregulate lipid synthesis (26). Activation of Nrf2 by apigenin neutralizes the activation of PPARγ to regulate lipid metabolism in liver (27).

(2) *Regulation of SREBP family.* Apigenin treatment inhibits lipid homeostasis by the sterol regulatory element-binding protein (SREBP) family. Apigenin significantly decrease lipid accumulation, total intracellular cholesterol (TC), and intracellular triglyceride (TG) levels *via* the AMPK-SREBP-1/2 (sterol regulatory element-binding protein-1/2) axis in HepG2 cells. Apigenin-induced activation of AMPK downregulates the levels of SREBP-1 and SREBP-2 to reduce the synthesis of cholesterol, fatty acids, and triglycerides in the liver. The inhibition of 3-hydroxy-3-methylglutaryl CoA reductase (HMGCR), which is the downstream target gene of SREBP-1 and FAS, the downstream target gene of SREBP-2 also regulates fatty acid and cholesterol synthesis (28).

(3) *Activation of genes related to fatty acid oxidation and cholesterol homeostasis*. Other genes related to fatty acid oxidation and cholesterol homeostasis in the liver, such as short/branched-chain acyl-CoA dehydrogenase (ASADSB), enoyl-CoA-hydratase and 3-hydroxyacyl-CoA dehydrogenase (EHHADH), Niemann-Pick type C 2 (NPC2) (29), HMG-CoA reductase (HMG-CoAR), low-density lipoprotein receptor (LDL-R), and cytochrome P450 family 7 subfamily A member 1 (CYP7A1) (30) have been reported to increase with apigenin treatment. In contrast, genes related to lipogenesis, such as PPARγ, lipoprotein lipase (LPL), sterol regulatory element-binding transcription factor 1 (SREBF1), and diacylglycerol O-acyltransferase 2 (DGAT2) were decreased in the liver (29).

Obesity-induced oxidative stress and inflammation also aggravate the symptoms of cardiometabolic diseases, leading to multiple cellular disorders (31, 32). Current studies indicate that apigenin alleviates oxidative stress and inflammation by binding to PPARγ as an agonist to regulate M2 polarization with nuclear factor kappa-light-chain-enhancer of activated B cells (NF-κB) inhibition. Apigenin-induced PPARγ activation blocks p65 nuclear translocation. NF-κB activation is inhibited in adipose tissue macrophages, leading to an increase in M2 macrophage polarization. The anti-inflammatory effects of M2 macrophages alleviate the metabolic disorder caused by obesity-related inflammation. Meanwhile, cytokines such as IL-6, IL-1β, and TNF-α are suppressed by the inhibition of NF-κB signaling (33) following apigenin treatment. The protective effect of apigenin on adipocyte browning in the inflammatory environment is also mediated *via* p65/NF-κB pathway. The inflammatory environment suppresses adipocyte browning to reduce lipid metabolism (34). Apigenin suppresses p65 translocation into the nucleus to inhibit NF-κB activation and inflammatory markers in adipocytes to attenuate inflammation and suppress adipocyte browning (25, 35). Apigenin also plays a protective role in the inhibition of white-to-brown adipose tissue differentiation. The inflammation induced *via* activation of uncoupling protein 1 and PGE2 receptor 4 (EP4) activates the cyclooxygenase 2 (COX2)/prostaglandin E2 (PGE2) axis, resulting in conversion of white to brown adipose tissue to generate heat by excessive energy expenditure (36).

Studies reported that obesity leads to many health complications. First, obesity has been associated with gastrointestinal disorders, such as gastroesophageal reflux disease, irritable bowel syndrome, and dyspepsia (37, 38). Colon inflammation adversely affects enteric motor function, leading to gastrointestinal disorders (39). Apigenin reduces the levels of malondialdehyde (MDA), interleukin-6 (IL-6), and interleukin-1β (IL-1β), as well as eosinophil infiltration in colon tissue to alleviate inflammation. Further, apigenin regulates inducible nitric oxide synthase (iNOS) expression and substance P (SP) levels in high-fat-diet (HFD)-fed obese mice (40). SP is a neurotransmitter that stimulates the contraction of various intestinal tissues and is a neurokinin receptor 1 (41). Obesity induces the expression of SP leading to enhanced tachykinergic transmission in the enteric nervous system, resulting in abnormal colonic motor function. The suppression of SP by apigenin attenuates enteric motor dysfunctions (40). NO produced by iNOS may trigger inflammation and play a role in enteric nitrergic pathways (42). The downregulation of iNOS by apigenin attenuates inflammation and enteric motor dysfunction. The regulation of gut bacteria by apigenin also prevents colonic dysfunction in mice *via* modulation of NOD-like receptor family pyrin domain containing 6 (Nlrp6) (43). Nlrp6 is highly expressed in the intestine. Nlrp6 deficiency may lead to proliferation of *Prevotellaceae*, the gut bacteria found in patients with bowel diseases (44), by promoting Nlrp6 inflammasome, IL-18 secretion, and regulation of gut bacterial homeostasis. Further, apigenin improves intestinal dysbiosis *via* augmentation of *Akkermansia* and *Incertae Sedis* along with reduction of *Faecalibaculum* and *Dubosiella* at the genus level (45). Obesity has also been associated with sarcopenia (46). Obesity-induced muscle atrophy also contributes to impaired glucose and lipid homeostasis, proinflammatory responses, and inflammation-induced mitochondrial dysfunction (47). Apigenin ameliorates skeletal muscle atrophy by enhancing mitochondrial function in an obese mouse model exposed to HFD and in C2C12 cells. Apigenin treatment upregulated mitochondria-related genes, including peroxisome proliferator-activated receptor-γ coactivator-1α (PGC1α), mt-TFAM (transcription factor of PGC1α), cytochrome C, and somatic cytochrome C (CyCs) following the activation of AMPK. Such upregulation is essential for initiation of mitochondrial biogenesis and improved mitochondrial function alleviate obesity-induced skeletal muscle atrophy (48, 49).

In summary, apigenin alleviates obesity and its complications *via* a variety of mechanisms including inhibition of appetite, glucose signaling pathways and lipid metabolism. It also regulates the intestinal microbiome, enhances mitochondrial function and attenuates inflammation and oxidative stress. The aforementioned experimental approaches and mechanisms underlying the effects of apigenin on obesity are listed in Table 1. Dosages and duration of apigenin treatment *in vivo* and *in vitro* are also listed in Table 1.

**TABLE 1:** Experiment designs and effects of apigenin on obesity and lipid metabolism (*in vivo* and *in vitro*).

Study design	Experiment models	Dose	Duration	Administration route	Source	Mechanisms	Reference
*In vivo* experiment	C57BL/6J mice (high fat diet)	10 mg/kg	48 h	Intraperitoneal injection (after modeling)	Seeds of *Perilla frutescens Britton* var *crispa* (Benth.)	Increase of POMC and CART expression to inhibit food intake	Myoung et al. (14)
	C57BL/6J mice (high fat diet)	10, 30, and 50 mg/kg	21 days	Intraperitoneal injection (after modeling)	Commercial	PPARγ activation to suppress NF-κB expression, leading to M2 polarization	Feng et al. (33)
	C57BL/6J ob/ob mice	30 mg/kg					
	C57BL/6J mice (high fat diet)	0.005%-supplemented (w/w)	16 weeks	Food intake (during modeling)	Commercial	Increase of expressions of fatty acid oxidation related genes, decrease of expressions of lipogenetic genes	Jung et al. (29)
	C57BL/6J mice (high fat diet)	30 mg/kg	3 weeks	Intraperitoneal injection (after modeling)	Commercial	Inhibition of PPARγ expression and activation of Nrf2	Feng et al. (27)
	ICR mice (high fat diet)	Not mentioned	28 days	Intragastric injection (during modeling)	Commercial	Decrease of blood fat, reduced animal weight, and reduced total cholesterol, triglyceride and low-density lipoprotein cholesterol	Zhang et al. (30)
	C57BL/6J mice (high fat diet)	10 mg/kg	8 weeks	Oral gavage (during modeling)	Commercial	Decrease of MDA, IL-6, IL-1β, SP, and iNOS expression	Gentile et al. (40)
	C57BL/6J mice (high fat diet)	0.04%-supplemented (w/w)	12 weeks	Food intake (during modeling)	Commercial	Activation of lipolysis and reduction of obesity-induced inflammation	Sun and Qu, (25)
	C57BL/6J mice (high fat diet)	15 and 30 mg/kg	13 days	Subcutaneous injection (after modeling)	Commercial	Decrease of STAT3, CD36 and PPARγ expression	Su et al. (16)
*In vitro* experiment	N29-2 neuronal cells	0.2, 1, and 5 μM	6 h	−	Seeds of *Perilla frutescens Britton* var *crispa* (Benth.)	Increase of POMC and CART expression	Myoung et al. (14)
	Human SHSY5Y cells						
	3T3-L1 cells	1, 10, and 50 μM	2 days	−	Commercial	AMPK activation to inhibit PPARγ expression	Ono and Fujimori (17)
	3T3-L1 cells	40 μM	4 days	−	Commercial	Decrease of pancreas lipase activity and preadipocyte differentiation	Guo et al. (23)
	ANA cells, RAW264.7 cells	7.5 μM	24 h	−	Commercial	M1/M2 polarization	Feng et al. (33)
	Human mesenchymal stem cells (hMSCs)	1, 10, and 25 μM	2 days	−	Commercial	Increase of atgl expression and decrease of fas expression	Gómez-Zorita et al. (24)
	THP-1 cells	Not mentioned	48 h	−	Commercial	Promotion of the efflux rate of [3H] cholesterol, increase of the activity of SOD and the amount of NO	Zhang et al. (30)
	HUVEC, VSC						
	Hep1-6 cells	0.2–64 μM	24 h	−	Commercial	Inhibition of PPARγ expression and activation of Nrf2	Feng et al. (27)
	HepG2 cells	0–1280 μM	24 h	−	Commercial	Activation of lipolysis and reduction of obesity-induced inflammation	Sun and Qu (25)
	Human adipose-derived stem cells (hASCs)	10 μM	48 h	−	Commercial	Activation of COX2/PGE2 axis to inhibit inflammation induced adipocyte browning	Okla et al. (35)
	3T3-L1 cells	50 and 100 μM	10 days	−	Commercial	Decrease of PPARγ	Su et al. (16)

*POMC, pro-opiomelanocortin; CART, cocaine- and amphetamine-related transcript; Nrf2, nuclear factor erythroid 2–related factor 2; MDA, malondialdehyde; SP, substance P; iNOS, inducible nitric oxide synthase; STAT3, signal transducer and activator of the transcription 3; CD36, cluster of differentiation 36; AMPK, 5′-Adenosine monophosphate-activated protein kinase; COX2, cyclooxygenase 2; PGE2, prostaglandin E2; atgl, adipose triglyceride lipase; fas, fatty acid synthase.*

### Protective Role of Apigenin in Diabetes

Diabetes also plays an important role in cardiometabolic disease (50–52). Several studies investigating the effects of apigenin on type 2 diabetes mellitus (T2DM) report decreased insulin resistance, reduced abnormal glycolipid metabolism, and alleviation of oxidative stress (53, 54).

Insulin resistance plays a significant role in the pathophysiology of T2DM (55). Insulin resistance adversely affects glycometabolism in insulin-targeted organs and tissues (54). Abnormal glycolipid metabolism is a typical clinical manifestation in patients with T2DM (56). Apigenin alleviates insulin resistance and glycolipid metabolic disorders *via* following mechanisms:

(1) *Inhibition of insulin receptor kinase.* Apigenin inhibits tyrosine nitration of the insulin receptor kinase domain leading to alleviation of insulin resistance. Tyrosine nitration of IRβ (intracellular β subunits of the insulin receptor) may lead to decreased tyrosine phosphorylation, resulting in impaired insulin signal transduction in HFD mice (57). *In vitro* studies showed that apigenin decreases the Cu^2+^-catalyzed insulin receptor kinase domain fragment KK-1 and inhibits the formation of 3,3′-dityrosine (58).

(2) *Regulation of miRNAs.* Apigenin regulates miRNAs, which are associated with insulin resistance and glucose homeostasis. *In vitro* experiments involving Huh7 cells and *in vivo* studies investigating miR103 transgenic mice validates apigenin-mediated inhibition of the phosphorylation of transactivating response RNA-binding proteins (TRBP). Additionally, miRNA-generating complexes inhibited, leading to suppression of precursor miRNA103 maturation expressed in liver and fat, resulting in insulin resistance and impaired glucose metabolism and homeostasis (59, 60). Thus, apigenin-induced suppression of miRNA103 alleviates glucose intolerance (60).

(3) *Upregulation of GLUT4/AMPK signaling.* Apigenin extracted from *Sophora davidii (Franch.)* promotes glucose transporter 4 (GLUT4) expression and activates AMPK phosphorylation in L6 cells and insulin target tissues in KK-Ay mice (61). In insulin target tissues such as liver and fat, the upregulation of GLUT4 and the activation of AMPK facilitates glucose utilization to ameliorate insulin resistance (62, 63).

(4) *Inhibition of*α*-amylase.* Several studies reported that apigenin decreases the inhibition of α-amylase in Kunming mice, thus reducing the digestion of dietary carbohydrates (64). The digestive enzyme α-amylase hydrolyzes dietary carbohydrates into disaccharides and polysaccharides (65). Inhibition of the digestion of dietary carbohydrates delays glucose absorption and blocks the progression of T2DM. Therefore, the inhibition of α-amylase by apigenin ameliorates T2DM (64).

Oxidative stress also triggers β-cell dysfunction, impaired glucose tolerance, and insulin resistance (66). The production of reactive oxygen species (ROS) in oxidative stress exacerbates the progression of T2DM and related complications. Apigenin treatment mitigates oxidative stress and intracellular ROS production *via* following mechanisms:

(1) *Decreased ROS production.* Apigenin pre-treatment of streptozocin (STZ)-treated RINm5F pancreatic β cells ameliorates STZ-induced intracellular ROS production, as well as DNA damage, lipid peroxidation, and apoptosis. Apigenin pre-treatment upregulates the expression of antioxidant enzymes, such as superoxide dismutase (SOD), catalase (CAT), and glutathione peroxidase (GSH-Px) in RINm5F pancreatic β cells and diabetic rats (67). SOD catalyzes the conversion of superoxide radicals (O_2–_) to molecular oxygen (O_2_) and hydrogen peroxide (H_2_O_2_), resulting in a protective effect against ROS in cells (68). Catalase is a highly specific enzyme that catalyzes the decomposition of hydrogen peroxide into water and molecular oxygen (69). GSH-Px is a cytosolic enzyme that catalyzes the reduction of hydrogen peroxide and lipid peroxides by glutathione, releasing water, oxygen, and alcohol (70). These three enzymes are indispensable in defending against free radicals (71).

(2) *Inhibition of AGE.* Apigenin inhibits the formation of advanced glycation end products (AGEs) and thereby alleviates oxidative stress (72). AGE-mediated damage leads to altered protein structure and functions *via* cross-linking between molecules *via* the receptor for AGEs (RAGE). AGEs increase ROS formation and damage anti-oxidant systems (73). Apigenin treatment of human blood plasma proteins *in vitro* reduced the levels of AGEs (72).

(3) *Regulation of Keap1-Nrf2 signaling*. The anti-oxidant function of apigenin is mediated *via* the Kelch-like ECH-associated protein 1 (Keap1)-Nrf2 axis targeting liver tissues to alleviate oxidative stress (74). Nrf2 is a primary transcription factor interacting with the anti-oxidant response element (ARE) to regulate antioxidant protein expression. Keap1 is the specific repressor of Nrf2, which acts as an adaptor protein of the Cullin3-based ubiquitin E3 ligase complex to facilitate the ubiquitination and subsequent proteolysis of Nrf2 (74), acting as a sensor of oxidative stress (75, 76). Apigenin occupies the Nrf2-binding site to prevent the binding between Keap1 and Nrf2 and thereby promotes nuclear translocation of Nrf2, thus facilitating its anti-oxidant function (74).

In addition, persistent inflammation leads to pathogenesis of diabetes (77). Apigenin significantly prevents mitogen-activated protein kinase activation (MAPK) from inhibiting inflammation (NF-κB-TNF-α axis) and apoptosis (increased expression of Bcl-2 and decreased Bax and caspase-3) in diabetic rats (78).

Currently, the apigenin-mediated regulation of blood glucose homeostasis can be summarized as follows: regulating the key enzymes and improving oxidative stress as well as inflammation. A detailed summary of the studies discussed above and the proposed mechanisms of apigenin-mediated effects in diabetes are presented in Table 2. Dosages and duration of apigenin treatments *in vivo* and *in vitro* are also listed in Table 2.

**TABLE 2:** Experiment designs and effects of apigenin on diabetes (*in vivo* and *in vitro*).

Study design	Experiment models	Dose	Duration	Administration route	Source	Mechanisms	Reference
*In vivo* experiment	miRNA103 transgenic mice	40 mg/kg	14 days	Intraperitoneal injection (after modeling)	Commercial	Inhibition of miRNA103 maturation	Ohno et al. (60)
	Wistar rats	10, 20, and 40 mg/kg	21 days	Intraperitoneal injection (after modeling)	Commercial	decrease of MDA content, increase of SOD activity and GSH level	Mao et al. (67)
	Sprague–Dawley rats	50 and 100 mg/kg	6 weeks	Oral gavage (after modeling)	Commercial	Inhibition of NF-κB activation and ICAM-1 mRNA expression	Ren et al. (97)
	C57BL/6J mice (high fat diet)	0.005% (w/w)	16 weeks	Food intake (during modeling)	Commercial	Upregulated expression of genes regulating fatty acid oxidation, TCA cycle and cholesterol homeostasis, downregulated expression of lipogenic genes in the liver	Jung et al. (29)
	C57BL/6J mice (high fructose diet)	50 mg/kg	4 weeks	oral gavage (during modeling)	Commercial	Inhibition of binding of Keap1 to Nrf2 to in increase the expressions of anti-oxidative genes	Yang et al. (126)
*In vitro* experiment	Huh7 cells	10 μM	24 h	−	Commercial	Inhibition of miRNA103 maturation	Ohno et al. (60)
	Hep3B cells, U-2 OS cells	30 μM	16 h	−	Commercial	Rapid intracellular translocation of FOXO1, downregulation of PEPCK, G6Pc, FASN and ACC, inhibition of the PKB/AKT-signaling pathway	Bumke-Vogt et al. (134)
	HepG2 cells	20 μM					
	HEK cells	20 μM					
	RINm5F rat pancreatic β cells	5 μM	1 h	−	Commercial	Reduction of intracellular ROS production, alleviation of DNA damage, lipid peroxidation, cell apoptosis of pancreatic beta cells, the loss of antioxidant enzymes	Wang et al. (71)
	Inhibition of apigenin against pancreatic α-Amylase	400 μM	10 min	−	Commercial	Inhibition against α-Amylase	Zhang et al. (64)
	H9c2 cells	1, 3, and 10 μM	20 h	−	Commercial	Inhibition of HIF-1α to improve abnormal glucolipid metabolism	Zhu et al. (105)
	Detection of Tyr phosphorylation: KK-1	40 μM	6 h	−	Commercial	Inhibition of tyrosine nitration of the insulin receptor kinase domain to alleviate insulin resistance	Fang et al. (58)

*Keap1, Kelch-like ECH-associated protein 1; Nrf2, nuclear factor erythroid 2–related factor 2; 2-NBDG, 2-[N-(7-Nitrobenz-2-oxa-1,3-diazol-4-yl)amino]-2-deoxy-D-glucose; ROS, reactive oxygen species; PKCβII, protein kinase C βII; HIF-1α, hypoxia-inducible factor 1 alpha.*

### Protective Role of Apigenin in Hypertension

Hypertension plays a central role in cardiometabolic diseases (79), which is prevalent in almost 80% of patients with metabolic syndrome (80). Recent studies reported that apigenin improves hypertension *via* attenuation of oxidative stress and recovery of mitochondrial dysfunction.

Apigenin plays a protective role in hypertension by alleviating oxidative stress. Apigenin can significantly restore normal blood pressure and reverse renal damage in cyclosporine-induced hypertensive Sprague-Dawley rats by decreasing lipid hydroperoxides and increasing anti-oxidant levels (81). Apigenin also controls elevated blood pressure in N-nitro-L-arginine methylester-induced hypertensive Sprague-Dawley rats by improving NO bioavailability, attenuating oxidative stress, and reducing vascular damage (82).

Apigenin also regulates pulmonary hypertension (PH). Mitochondrial dysfunction plays a vital role in PH, it may lead to the imbalance of ion homeostasis and downregulation of enzymes in apoptosis (83). Apigenin activates mitochondria-dependent apoptosis *via* hypoxia-inducible factor 1α (HIF-1α)-KV1.5 channel pathway. The inhibition of HIF-1α by apigenin upregulates the expression of KV1.5 channels to restore mitochondrial function, thereby attenuating PH (84).

Apigenin has also been reported to diminish the complications induced by hypertension, such as renal damage and fibrosis due to abnormal collagen accumulation in kidneys (85). Apigenin significantly attenuated hypertension and renal fibrosis in deoxycorticosterone acetate (DOCA)-salt-induced hypertensive rats (86). Apigenin activates transient receptor potential vanilloid 4 (TRPV4), a non-selective cation channel widely expressed in the kidney. Ca^2+^ influx is then promoted in vascular endothelium and smooth muscle to induce vasodilation (87) and activation of the AMPK/SIRT1 signaling pathway to inhibit the TGF-β1 and Smad-2/3 signaling pathway (Sma and Mad proteins from *Caenorhabditis elegans* and *Drosophila*, respectively). This inhibition stimulates cellular transformation into fibroblasts and increases the synthesis of matrix proteins to induce renal fibrosis (86, 88, 89). Thus, apigenin alleviates renal fibrosis and structural and functional damage.

Current evidence suggests that apigenin decreases blood pressure mainly *via* improved NO bioactivity and oxidative stress, regulation of apoptosis-related mitochondrial genes and promotion of vasodilation in vascular endothelium. Experimental studies and mechanisms of action involving apigenin in hypertension are listed in Table 3. Experimental dosages and durations of apigenin treatment *in vivo* and *in vitro* are listed in Table 3.

**TABLE 3:** Experiment designs and effects of apigenin on hypertension (*in vivo* and *in vitro*).

Study design	Experiment models	Dose	Duration	Administration route	Sources	Mechanisms	Reference
*In vivo* experiment	Sprague-Dawley rats (cyclo-sporine induced)	10, 15, and 20 mg/kg	21 days	oral gavage (during modeling)	Commercial	Reduction of the lipid hydroperoxides and increase of the total antioxidant levels	Haleagrahara et al. (81)
	Sprague-Dawley rats (DOCA-salt treated)	0.2%-supplement (w/w)	4 weeks	Food intake (during modeling)	Commercial	TRPV4-mediated activation of AMPK/SIRT1 and inhibition of the TGF-β1/Smad2/3 signaling pathway	Wei et al. (86)
	Sprague Dawley rats (L-NAME induced)	1.44 mg/kg	6 weeks	Drinking water (during modeling)	Commercial	Improvement of NO bioavailability and endothelial and vascular function, alleviation of oxidative stress	Paredes et al. (82)
	Sprague-Dawley rats	50 and 100 mg/kg	4 weeks	intragastric administration (during modeling)	Commercial	Modulation of HIF-1α signaling, the induction of apoptosis factors Bax, Bcl-2, cleaved caspase 3, and cleaved caspase 9	He et al. (84)
*In vitro* experiment	HBZY-1 cells, M1CCD cells	5 μM	24 h	−	Commercial	TRPV4-mediated activation of AMPK/SIRT1 and inhibition of the TGF-β1/Smad2/3 signaling pathway	Wei et al. (86)

*HIF-1α, hypoxia-inducible factor 1 alpha; CPT-1, carnitine palmitoyltransferase 1; PDK-4, pyruvate dehydrogenase kinase 4; GPAT, glycerol-3-phosphate acyltransferase; GLUT-4, glucose transporter 4; DOCA, deoxycorticosterone acetate; TRPV4, transient receptor potential vanilloid 4; AMPK, 5′-Adenosine monophosphate-activated protein kinase; SIRT1, Sirtuin 1; Smad, Sma and Mad proteins from Caenorhabditis elegans and Drosophila, respectively; Bax, Bcl-2 associated X; Bcl-2, B-cell lymphoma.*

### Protective Role of Apigenin in Cardiovascular Diseases

Apigenin prevents cardiovascular diseases *via* antioxidant and anti-apoptotic mechanisms in vascular endothelial cells and cardiomyocytes.

Vascular endothelial dysfunction is a major mediator in cardiovascular diseases (90). Abnormal glucose metabolism and oxidative stress in vascular endothelial cells may lead to vascular endothelial dysfunction. Several studies have discussed the ameliorative effect of apigenin on endothelial dysfunction. First, apigenin increases apelin expression to rescue endothelial dysfunction. Apelin is an endogenous ligand for the G-protein-coupled APJ receptor expressed in the cardiovascular system. It increases glucose uptake and SOD activity, reversing the impaired glucose metabolism and homeostasis and the severe oxidative stress in human endothelial cells (91–94). Oxidative stress in endothelial cells leads to endothelial dysfunction and angiogenesis (92). The suppression of apelin in human endothelial cells can be reversed by apigenin treatment. Second, apigenin inhibits NF-κB-associated signaling pathways and suppresses intercellular adhesion molecule-1 (ICAM-1) expression in vascular endothelial dysfunction. ICAM-1 is a cell surface receptor that binds lymphocyte function-associated antigen 1 (LFA-1), mediating the interaction between keratinocytes and leukocytes (95). ICAM-1 plays an essential role in controlling abnormal inflammatory infiltration, adhesion, and migration (96). Apigenin inhibits NF-κB activation to improve NO production and SOD activity in endothelial cells and suppress ICAM-1 expression in human vascular endothelial cells (HUVECs) (97). Third, apigenin inactivates the PI3K (phosphoinositide-3-kinase) Akt (protein kinase B) axis in HUVECs during vascular endothelial dysfunction. The PI3K/Akt axis is an essential pathway in the pathogenesis of cardiovascular complications in T2DM (98). Apigenin treatment inhibited the phosphorylation of Akt-residues Ser473 and Thr308 to prevent vascular endothelial dysfunction (99). Finally, apigenin decreased ROS and improved NO levels to alleviate vascular endothelial dysfunction induced by mitochondria-dependent apoptosis *via* inhibition of protein kinase C βII (PKCβII) phosphorylation. PKCβII promotes oxidative stress, ROS production and mitochondria-dependent apoptosis in vascular endothelial dysfunction (100, 101). Apigenin treatment upregulated the expression of the anti-apoptotic gene, B-cell lymphoma-2 (Bcl-2), while the pro-apoptotic gene, Bcl-2 associated X (Bax), was downregulated, resulting in attenuation of mitochondria-dependent apoptosis in endothelial cells (102).

Further, cardiac hypertrophy is another manifestation of cardiovascular diseases (103). Current evidence suggests that abnormal glycolipid metabolism and overexpression of HIF-1α in cardiac cells causes cardiac hypertrophy (104). *In vivo* and *in vitro* experimental data suggest that apigenin alleviates cardiac hypertrophy *via* suppression of HIF-1α, thereby reversing the expression of PPARα/γ and target genes including glycerol-3-phosphate acyltransferase (GPAT), glucose transporter 4 (GLUT-4), carnitine palmitoyltransferase 1 (CPT-1) and pyruvate dehydrogenase kinase 4 (PDK-4). Downregulation of GLUT4 and upregulation of PDK-4 can inhibit excessive glucose intake and oxidation, preventing abnormal glucose metabolism. Downregulation of GPAT and upregulation of CPT-1 decreases the rate of triglyceride synthesis and augments fatty acid oxidation, thereby improving lipid metabolism (105, 106). Thus, the hypoxic myocardial energy utilization (107–110) can be reversed.

In summary, apigenin can ameliorate cardiovascular diseases *via* reduction of oxidative stress and mitochondria-dependent apoptosis in vascular endothelial cells as well as regulation of glucose and lipid metabolism in cardiomyocytes. The experimental studies and mechanisms of action involving apigenin in hypertension are presented in Table 4. Experimental dosages and duration of apigenin treatment *in vivo* and *in vitro* are also listed in Table 4.

**TABLE 4:** Experiment designs and effects of apigenin on cardiovascular diseases (*in vivo* and *in vitro*).

Study design	Experiment models	Dose	Duration	Administration route	Sources	Mechanisms	Reference
*In vivo* experiment	Sprague-Dawley rats (left renal artery ligation)	50 and 100 mg/kg	8 weeks	oral gavage (after modeling)	Commercial	Down-regulation of myocardial HIF-1α expression, increase of the expressions of myocardial PPARα, CPT-1 and PDK-4, decrease of expressions of myocardial PPARγ, GPAT and GLUT-4	Zhu et al. (106)
*In vitro* experiment	ISO-HAS cells	30 μM	0–24 h	−	Commercial	Increase of Apelin to rescue endothelial dysfunction	Yamagata et al. (92)
	HUVEC	3 and 30 μM	30 min	−	Commercial	Inactivation of PI3K/Akt axis to mediate vascular endothelial dysfunction	Yu et al. (98)
	HUVEC	3 and 30 μM	48 and 72 h	−	Commercial	Inhibition of PKCβII phosphorylation and regulation of apoptosis-dependent genes	Qin et al. (102)
	HUVEC	3 and 30 μM	30 min	−	Commercial	Inhibition of NF-κB activation to improve NO and SOD activity, suppression of ICAM-1	Ren et al. (97)

### Apigenin Analogs and Their Effects on Alleviating Cardiometabolic Diseases

Apigenin analogs are derived from the basic flavonoid skeleton *via* hydroxyl group substitution, glycosylation, hydroxylation, and methylation (111, 112). In plants, apigenin is stored as glycosides such as apigenin 7-O-apioglucoside in celery and parsley (113, 114) and apigenin 8-C-glucoside isolated from bamboo leaves (115). Several apigenin analogs carry the basic flavonoid skeleton similar to apigenin and exhibit biological activity in cardiometabolic diseases.

(1) *Apiin*. Apiin (apigenin 7-O-apioglucoside) is derived from celery and exhibits anti-adipogenic and anti-obesity effects in HFD mice *via* the AMPK/PPARγ axis (20), similar to apigenin. Apiin also alleviates insulin resistance in HFD mice *via* downregulation of glucogenic genes, PEPCK (phosphoenolpyruvate carboxykinase) and G6Pase (glucose-6-phosphatase) in the liver, and promotion of glycogen synthesis *via* inhibition of glycogen synthase phosphorylation and induction of GSK3β (glycogen synthase kinase3β) phosphorylation (116).

(2) *Apigetrin.* Apigetrin (apigenin 7-glucoside) ameliorated pancreatic β cell damage *via* reduction of endoplasmic reticulum (ER) stress in RINm5F cells *via* the regulation of ER stress biomarkers, such as upregulation of CCAAT/enhancer-binding protein homologous protein (C/EBP), induction of spliced X-box binding protein 1 (XBP1), phosphorylation of protein kinase RNA-like ER kinase (PERK) and eukaryotic initiation factor 2α (eIF2alpha), and cleavage of caspase-12 (117).

(3) *Vitexin*. Vitexin (apigenin 8-C-glucoside) regulates lipid metabolism *via* AMPK-mediated pathway in 3T3-L1 cells (118) *in vitro* and the liver of HFD mice (119) *in vivo* to alleviate obesity and non-alcoholic fatty liver disease. Vitexin also protects pancreatic β-cells *via* inhibition of high mobility group box 1 (HMGB1) (120), which is released from damaged pancreatic β-cells and induces inflammation in LPS (lipopolysaccharide)-induced rats and LPS-treated INS-cells.

(4) *Acacetin*. Acacetin (4′-methoxy 5,7-dihydroxyflavone) suppress adipogenesis in 3T3-L1 cells and HFD mice *via* upregulation of SIRT1 expression and AMPK phosphorylation (121). Acacetin also increases glucose uptake by enhancing GLUT4 translocation to the plasma membrane *via* the CaMKII-AMPK pathway by increasing intracellular calcium concentrations in L6 and HepG2 cells (122). In addition to regulating glycometabolism, acacetin alleviates endothelial dysfunction in insulin-resistant rats by inhibiting the release of inflammatory factors, such as NF-κB and IL-1β, and improving vasodilatory function *via* the estrogen signaling pathway (123).

(5) *Apigenin 7, 4*′*-dimethyl ether*. Apigenin 7, 4′-dimethyl ether (ADE) enhances glucose uptake in L6 cells and inhibits α-glucosidase enzyme (124), which releases glucose to form glycolipid and glycopeptide *via* hydrolyzation of α-glycosidic bonds from the non-reducing ends of oligosaccharide substrates and transfer of free glucose residues to another carbohydrate substrate.

(6) 8-(6″-umbelliferyl)-apigenin. 8-(6″-umbelliferyl)-apigenin promotes glucose uptake in 3T3-L1 cells, indicating improved glucose consumption (125).

## Potential Signaling Pathways Mediated by Apigenin for Amelioration of Cardiometabolic Disease in Different Cell Types

**FIGURE 2:**
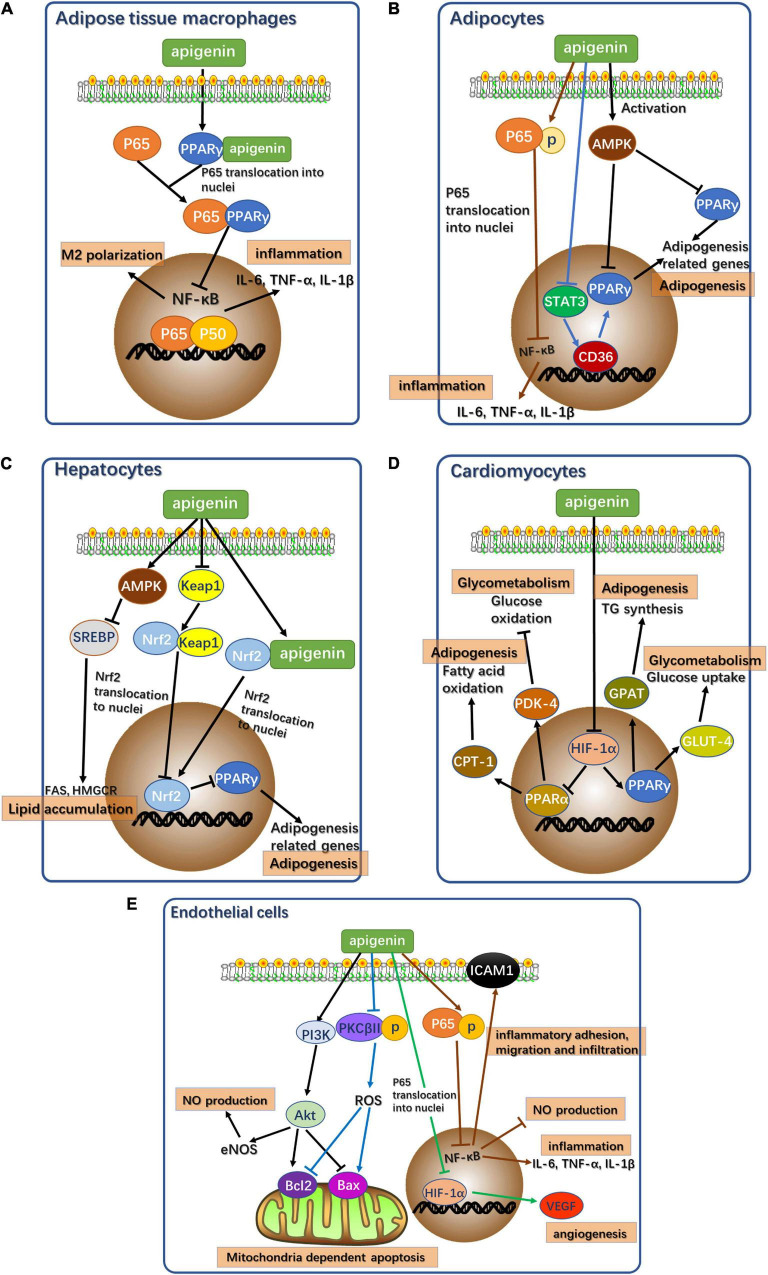
Potential signaling pathways of apigenin affecting cardiometabolic diseases in different types of cells. **(A)** Potential signaling pathways of apigenin affecting cardiometabolic diseases in adipose tissue macrophages. p65: RelA, NF-κB component. P50: p50 NF-κB component. **(B)** Potential signaling pathways of apigenin affecting cardiometabolic diseases in adipocytes. AMPK, AMP-activated protein kinase. p65: RelA, NF-κB component. STAT3, Signal transducer and activator of transcription 3. **(C)** Potential signaling pathways of apigenin affecting cardiometabolic diseases in hepatocytes. AMPK, AMP-activated protein kinase. SREBP, sterol regulatory element-binding proteins. FAS, fatty acid synthase. HMGCR, 3-Hydroxy-3-Methylglutaryl-Coenzyme A Reductase. Keap1, Kelch like ECH associated protein 1. Nrf2, NF-E2-related factor 2. **(D)** Potential signaling pathways of apigenin affecting cardiometabolic diseases in cardiomyocytes. HIF-1α, hypoxia inducible factor 1 subunit alpha; CPT1, carnitine palmitoyl transferase I; PDK4, pyruvate dehydrogenase kinase 4; GPAT, glycerol-3-phosphate acyltransferase; GLUT4, glucose transporter type 4. **(E)** Potential signaling pathways of apigenin affecting cardiometabolic diseases in endothelial cells. PI3K, Phosphoinositide 3-kinase; Akt, protein kinase B; eNOS, endothelial nitric oxide synthase; PKCβII, protein kinase C subunit β II; ROS, reactive oxygen species; Bcl-2, B-cell lymphoma-2; Bax, Bcl2 associated X; HIF-1α, hypoxia inducible factor 1 subunit alpha; VEGF, vascular endothelial growth factor; p65, RelA, NF-κB component.

In summary, the potential signaling pathways mediated by apigenin resulting in alleviation of cardiometabolic diseases in different cell types are illustrated in Figures 2A–E. Apigenin alleviates cardiometabolic diseases mainly by regulating glycolipid metabolism, oxidative stress, and oxidative stress-induced inflammation and apoptosis. Notably, apigenin plays contrasting roles in different types of cells. Apigenin acts as an agonist of PPARγ in adipose tissue macrophages. PPARγ binds to p65 to inhibit nuclear translocation to block NF-κB signaling pathway resulting in attenuation of inflammation (Figure 2A) (33). However, apigenin inhibits PPARγ expression in adipocytes, hepatocytes, and cardiomyocytes by acting as an antagonist (Figures 2B–D).

### Apigenin in Adipocytes

In adipocytes, apigenin acts as a functional regulator of lipid metabolism to reduce fat accumulation. Apigenin downregulates PPARγ expression by inhibiting the STAT3/CD36 axis (16) and the activation of AMPK (17). Apigenin directly induces p65 phosphorylation to prevent its nuclear translocation to ensure continued inhibition of NF-κB signaling in the absence of PPARγ as a mediator (25, 35).

### Apigenin in Hepatocytes

Since the liver is an important site of energy metabolism, apigenin is a potential mediator of glycolipid metabolism in hepatocytes. Apigenin acts as a PPARγ antagonist *via* direct activation of Nrf2 and indirect activation of Nrf2 *via* the Keap1-Nrf2 pathway (126). Additionally, apigenin activates AMPK to inhibit SREBP-1 and SPEBP-2 to regulate hepatic fatty acid oxidation and cholesterol synthesis (28).

### Apigenin in Cardiomyocytes

Apigenin treatment of cardiomyocytes regulates glucose and lipid metabolism to maintain normal cellular function. HIF-1α activation *via* apigenin regulates the PPAR family, leading to the appropriate regulation of downstream target genes related to glycolipid metabolism. Apigenin suppresses PPARγ expression *via* the activation of HIF-1α as an antagonist (105, 106). Meanwhile, the upregulation of HIF-1α following apigenin treatment increases PPARα expression (105, 106).

### Apigenin in Endothelial Cells

Apigenin plays a protective role in vascular endothelial dysfunction by regulating several signaling pathways in endothelial cells to alleviate oxidative stress, inflammation, and mitochondria-dependent apoptosis. The inhibition of the NF-κB signaling pathway with apigenin treatment also ameliorates inflammatory response in endothelial cells and increases NO production (97). NF-κB inhibition also suppresses the expression of ICAM-1 to improve abnormal inflammatory adhesion, migration, and infiltration, resulting in the alleviation of vascular endothelial dysfunction (97). In addition to NF-κB pathways, apigenin also activates the PI3K-Akt pathway and inhibits PKCβII activation by reducing oxidative stress and oxidative stress-related apoptosis in mitochondria (102). The expression of anti-apoptotic gene, Bcl-2, and the pro-apoptotic gene, Bax, in these two pathways reduces abnormal apoptosis. The activation of the PI3K-Akt pathway also promotes eNOS activity to restore NO levels and thereby attenuates oxidative stress (102). Apigenin treatment also mitigates angiogenesis induced by inflammation. HIF-1α inhibition by apigenin directly reduces the expression of vascular endothelial growth factor (VEGF) in angiogenesis, thus alleviating the angiogenesis induced by vascular dysfunction (127).

## Conclusion and Perspectives

**FIGURE 3:**
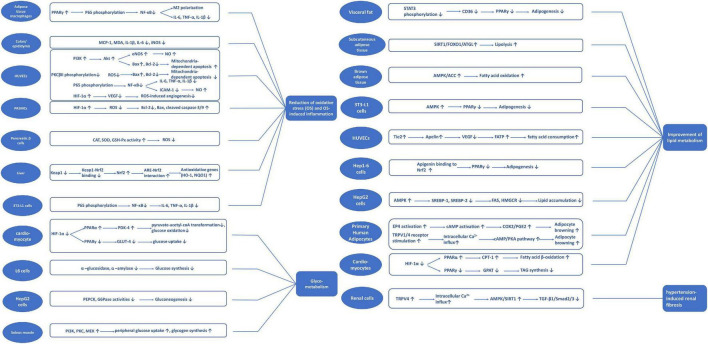
Potential signaling pathways classified with different mechanisms of apigenin on cardiometabolic diseases.

A review of studies investigating apigenin suggests critical biological mechanisms, including reducing oxidative stress and oxidative stress-induced inflammation and apoptosis, and improving glycolipid metabolism. Figure 3 summarizes the potential signaling pathways of apigenin underlying the protection against cardiometabolic diseases.

The molecular structure of apigenin suggests poor water solubility, chemical instability and moderate permeability, which prevent maximum bioavailability. Therefore, new delivery and design strategies have been formulated including the development of apigenin glycosides and acylated derivatives to enhance water solubility (128, 129). Apigenin-AuNP complex can be developed at room temperature at pH 10 to enhance the stability of apigenin in the body (130). Nano-apigenin using poly (lactic-co-glycolide) (PLGA) can also improve the bioactivity of apigenin (131). Pharmacokinetic and pharmacodynamic profiles of apigenin in rats and mice have been studied. The peak plasma concentration C_max_ and the time to reach the peak plasma concentration T_max_ were 1.07 ng/mL and 1 h, respectively, and the area under the concentration-time curve (AUC_0–24_) was 3.9 ng h/mL in mice (132). However, the bioavailability of apigenin in humans is still unknown. Further studies are needed to confirm the bioavailability and safety profile in humans.

In summary, the extensive review and validation of *in vitro* and *in vivo* evidence suggests that apigenin is a natural compound that can be used to protect against cardiometabolic diseases. Environment-wide association studies (EWAS) also indicate that apigenin is one of the protective factors in cardiovascular diseases at the population level (133). Further studies are required to establish the optimum dose of apigenin in alleviating cardiometabolic diseases in humans, developing a novel approach for clinical management of the disease.

## Author Contributions

XL designed this review, helped with writing and revising of the manuscript, and provided critical feedback. YX contributed to collecting and screening the literature, as well as summarizing the data, and then composed and revised the manuscript. HW reviewed the manuscript. All authors were involved in final approval of the submitted version.

## Conflict of Interest

The authors declare that the research was conducted in the absence of any commercial or financial relationships that could be construed as a potential conflict of interest.

## Publisher’s Note

All claims expressed in this article are solely those of the authors and do not necessarily represent those of their affiliated organizations, or those of the publisher, the editors and the reviewers. Any product that may be evaluated in this article, or claim that may be made by its manufacturer, is not guaranteed or endorsed by the publisher.
